# Does Chinese calligraphy therapy reduce neuropsychiatric symptoms: a systematic review and meta-analysis

**DOI:** 10.1186/s12888-018-1611-4

**Published:** 2018-03-07

**Authors:** Kuan-Yu Chu, Chih-Yang Huang, Wen-Chen Ouyang

**Affiliations:** 1 Taoyuan General Hospital, Taoyuan City, Taiwan; 2 Department of Fine Art & Culture Creative Design, Hua-Fan University, New Taipei City, Taiwan; 3 College of General studies, Yuan-Ze University, Taoyuan City, Taiwan; 4 Jianan Psychiatric Center, Tainan City, Taiwan; 5 Department of Psychiatry, College of Medicine, Kaohsiung Medical University, Kaohsiung, Taiwan

**Keywords:** Chinese calligraphy, Art therapy, Psychiatric disorders

## Abstract

**Background:**

There are currently no systematic reviews or meta-analyses of Chinese calligraphy therapy (CCT) to reduce neuropsychiatric symptoms. The aim of this systematic review and meta-analysis was to explore the efficacy of CCT for people with neuropsychiatric symptoms.

**Methods:**

We searched Chinese and English databases, including the Cochrane Central Register of Controlled Trials and Wanfang Data for relevant articles published between the earliest year available and December 2016. The search was limited to randomized controlled trials and controlled clinical studies and the associated keywords were “handwriting,” “Chinese calligraphy,” “Chinese calligraphy therapy,” “Calligraphy exercise,” and “Calligraphy training.” The 21 articles that met these criteria were used in the analysis. The Joanna Briggs Institute critical appraisal checklist was used to assess methodological quality.

**Results:**

CCT significantly reduced psychosis (10 studies, 965 subjects, standardized mean difference [SMD] = − 0.17, 95% confidence intervals [CI] [− 0.30, − 0.40], Z = 2.60, *p* < 0.01), anxiety symptoms (9 studies, 579 subjects, SMD = − 0.78, 95% CI [− 0.95, − 0.61], Z = 8.98, *p* < 0.001), and depressive symptoms (7 studies, 456 subjects, SMD = − 0.69, 95% CI [− 0.88, − 0.50], Z = 7.11, p < 0.001). CCT also significantly improved cognitive function (2 studies, 55 subjects, MD = 2.17, 95% CI [− 0.03, 4.38], Z = 1.93, *p* = 0.05) and neurofeedback (3 studies, 148 subjects, SMD = − 1.09, 95% CI [− 1.44, − 0.73], Z = 6.01, *p* < 0.001). The therapy also significantly reduced the positive psychopathological expression of schizophrenia symptoms (4 studies, 287 subjects, SMD = − 0.35, 95% CI [− 0.59, − 0.12], Z = 2.96, *p* = 0.003) and reduced the negative symptoms of schizophrenia (4 studies, 276 subjects, SMD = − 1.39, 95% CI [− 1.65, − 1.12], Z = 10.23, *p* < 0.001).

**Conclusions:**

CCT exerts a curative effect on neuropsychiatric symptoms, but the evidence remains insufficient. A large number of RCTs are needed to facilitate additional systematic reviews of evidence for CCT.

**Electronic supplementary material:**

The online version of this article (10.1186/s12888-018-1611-4) contains supplementary material, which is available to authorized users.

## Background

The term psychosis refers to a serious psychological disorder characterized by obvious and long-lasting abnormalities in understanding, emotion, cognition, behavior, and other psychological activities. Psychoses such as schizophrenia often involve cognitive impairment and comorbid anxiety and depression [1]. Pharmacological therapy often has a limited effect or produces side effects [2, 3]. Non-pharmacological therapies (such as psychotherapy, occupational therapy, and art therapy) for patients with psychiatric disorders are useful, adaptable, and potentially cost-effective approaches to improve outcomes and quality of life [4].

Chinese calligraphy therapy (CCT) is a branch of art therapy that involves visual–spatial patterning of characters. This type of calligraphy is more than an art therapy; in essence, it involves culture, health, behaviour treatment and rehabilitation. The art nature is only one of its varied roles and functions. It necessitates exercising motor control of the brush to follow specific character configurations based on a projection of the cognitive images of the characters [5]. CCT requires the use of a soft-tipped brush to reproduce Chinese glyphs. It combines physical, mental, and personal processes and integrates visual performance, spatial abilities, and cognitive planning [6].

Recent empirical studies have shown that the practice of calligraphy may improve behavioral and psychosomatic disorders and may have a therapeutic effect on attention and emotional stability [7]. CCT has been scientifically investigated within the contexts of psychology, cognitive science, and cognitive neuroscience, and the findings suggest that it can reduce neuropsychiatric symptoms [8].

There have been many systematic reviews of other art therapies, such as painting therapy, music therapy, and gardening therapy. However, there are no systematic reviews or meta-analyses of CCT’s effect in reducing neuropsychiatric symptoms. Thus, the aim of this systematic review and meta-analysis was to explore the efficacy of CCT for people with neuropsychiatric symptoms.

## Methods

### Search strategy

A systematic search for articles was made in December 2016 using the databases MEDLINE, EMBASE, PsycINFO, Cochrane Central Register of Controlled Trials, and Wanfang Data. We searched for keywords and/or controlled vocabulary, such as medical subject headings and Emtree terms. Keywords were “handwriting,” “Chinese calligraphy,” “Chinese calligraphy therapy,” “Calligraphy exercise,” and “Calligraphy training.”

### Study selection

We included in the analysis studies that met the following criteria: (1) randomized controlled trial (RCT), cohort study, or case-control study, (2) published in Chinese or English, (3) subjects were either healthy or psychiatric patients, (4) experimental group intervention measures for Chinese calligraphy; the control group intervention could be general care, health education, or no care, and (5) measurement of physiological or mental indicators. The exclusion criteria were as follows: (1) not relevant to Chinese calligraphy/calligraphy therapy, (2) commentary, case report, or a review article, (3) experimental interventions other than calligraphy treatment, (4) and lack of a control group and/or overlapping populations.

### Quality assessment

We used the guideline suggested by Queen’s Joanna Briggs Collaboration critical appraisal checklist, Version 4.0 [9]. This scale contains 10 appraisal criteria that assess whether the assignment to treatment groups was truly random, participants were blinded to treatment allocation, allocation to treatment groups was concealed from the allocator, the outcomes of people who withdrew were described and included in the analysis, those assessing the outcomes were blind to the treatment allocation, the control and treatment groups were comparable at entry, the groups were treated identically other than for the named interventions, the outcomes were measured in the same way for all groups, the outcomes were measured in a reliable way, and the statistical analysis was appropriate. Two researchers extracted information and screened the quality of the articles independently. A third researcher was used to determine the quality of the studies in cases where it was difficult to reach a consensus.

### Data analysis

Meta-analysis was performed using RevMan 5.3 software (Review Manager (RevMan) [Computer program]. Version 5.3. Copenhagen: The Nordic Cochrane Centre, The Cochrane Collaboration, 2014.). The mean and standard deviation of each group was calculated to determine the overall effect of the intervention. Chi-square was used to determine heterogeneity. In addition, I2 values were calculated to test the heterogeneity among the studies. When I2 was < 50%, a fixed-effects model was used to determine the homogeneity among studies [10]. However, if there were differences between the studies (such as study location, population, and intervention program), a random-effects model analysis was used to avoid underestimation of treatment variability. Odds ratios and mean differences were used to compare different measurement indexes, to obtain standardized mean differences (SMD), and to estimate the combined effect level. Based on the different psychiatric symptoms and diseases included in the studies, we divided the meta-analysis into six groups: group 1: psychosis, group 2: anxiety, group 3: depression, group 4: cognition, group 5: neurofeedback, and group 6: schizophrenia.

## Results

### Characteristics of the studies

**Fig. 1:**
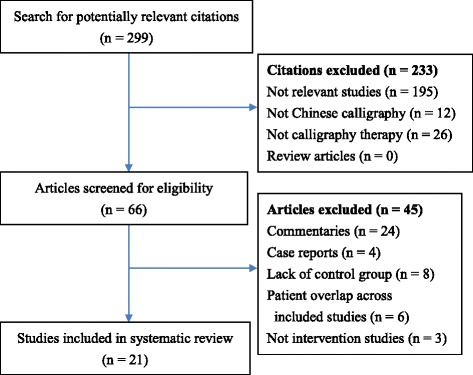
Flow diagram of study selection

Figure 1 shows the process of study selection. Our initial search strategy yielded 299 citations, 233 of which were ineligible based on our screening of titles and abstracts. Thus, we retrieved the full text of 66 studies. Of these, 24 were excluded because they were commentaries; 4 were case reports; 8 lacked a control group; 6 had study populations that overlapped with other included studies; and 3 did not meet the eligibility criteria because they were not intervention studies. Consequently, 21 eligible studies were analyzed.

**Fig. 2:**
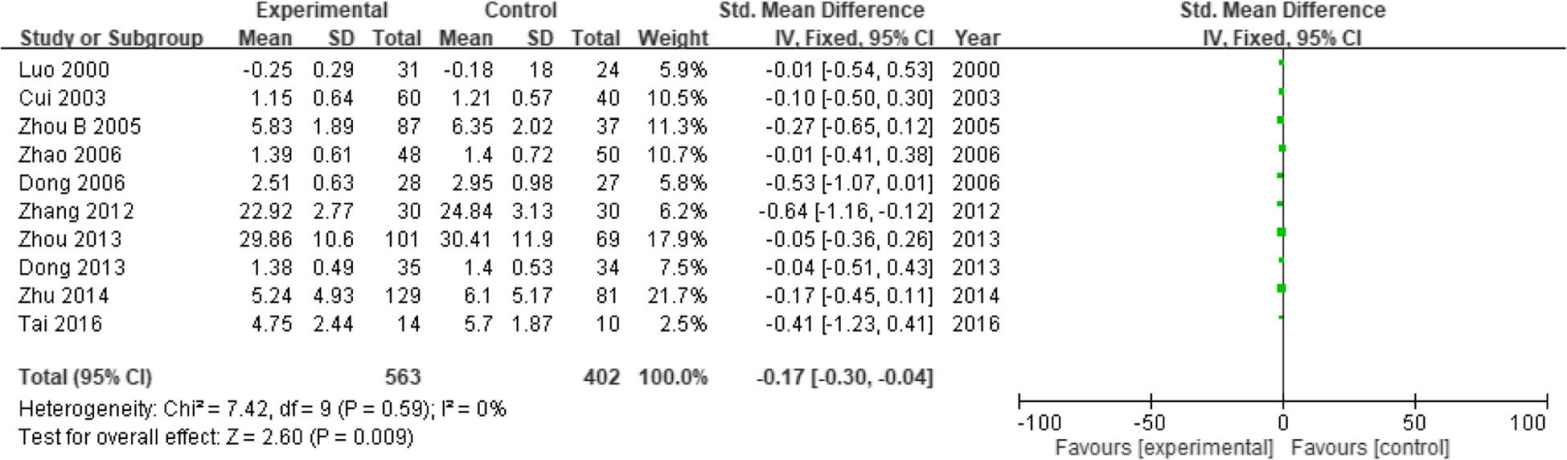
Forest plot of comparison: Experimental (Chinese calligraphy therapy, etc.) versus Control. Outcome: index of psychosis

**Fig. 3:**
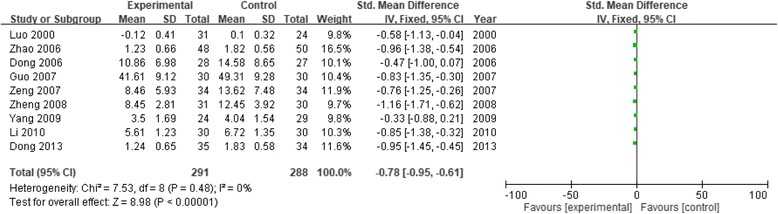
Forest plot of comparison: Experimental (Chinese calligraphy therapy, etc.) versus Control. Outcome: index of anxiety

**Fig. 4:**
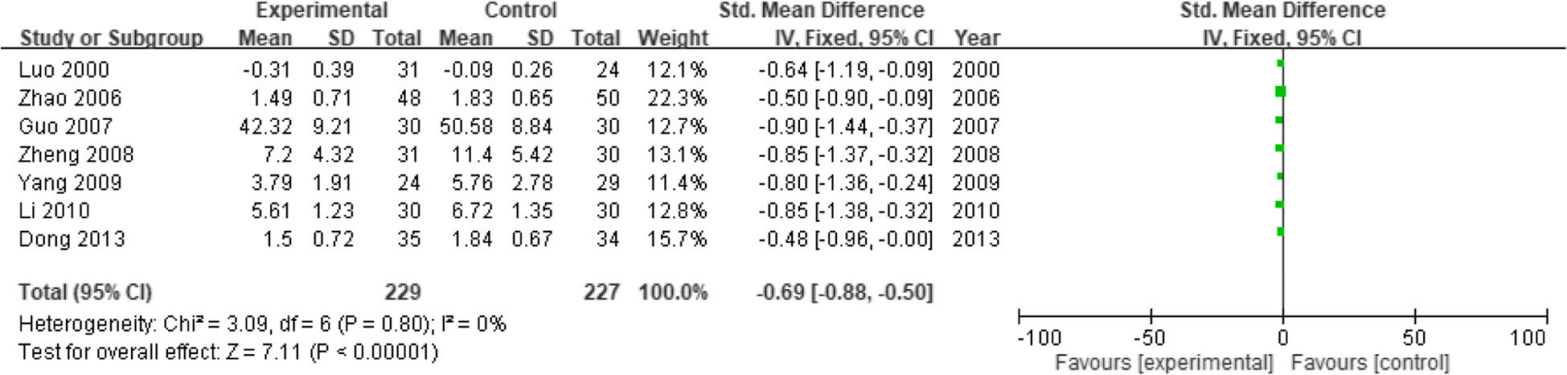
Forest plot of comparison: Experimental (Chinese calligraphy therapy, etc.) versus Control. Outcome: index of depression

**Fig. 5:**
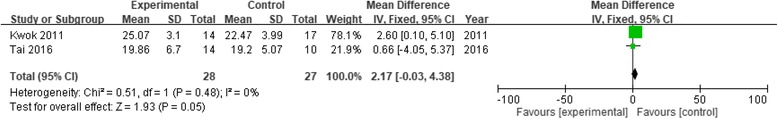
Forest plot of comparison: Experimental (Chinese calligraphy therapy, etc.) versus Control. Outcome: index of cognitive function

**Fig. 6:**
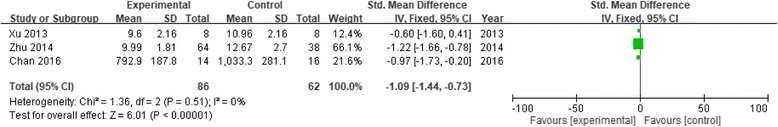
Forest plot of comparison: Experimental (Chinese calligraphy therapy, etc.) versus Control. Outcome: index of neurofeedback

Table 1 shows study characteristics and patient demographic data from each of the 21 studies included in the review. These studies were published between 2000 and 2016, and had sample sizes ranging from 16 to 224. Ten studies measured psychosis or general psychosis (the meta-analysis results and forest map are shown in Fig. 2). Nine studies on anxiety were analyzed; one study was removed [11] because the sensitivity analysis indicated large heterogeneity (Fig. 3). Seven studies on depression were analyzed (Fig. 4). Two studies on cognitive impairment were analyzed (Fig. 5) and three studies on neurofeedback were analyzed (Fig. 6).

**Table 1:** Characteristics of studies that fulfilled inclusion criteria

Study	Inclusion criteria	No. of patients	Treatment duration	Ages (yrs) (mean ± SD)	Main measurement scale	Intervention
Luo 2000 [14]	Military college students	P: 31 C: 24	30 days	23.9	SCL-90	Calligraphy
Cui 2003 [11]	Senior college students	P: 60 C: 40	1 year	P: 65.4 ± 5.25 C: 64.8 ± 8.46	SCL-90	Calligraphy and painting
Zhou B 2005 [15]	Grade 3 elementary school students	P: 87 C: 37	2 years	–	CPQ	Calligraphy
Zhou GQ 2005 [16]	Patients with schizophrenia	P: 15 C: 21	20 weeks	P: 49.67 ± 6.9 C: 53.33 ± 6.64	PANSS TESS	Calligraphy
Dong 2006 [17]	Patients with anxiety	P: 28 C: 27	8 weeks	P: 32.4 ± 7.8 C: 30.5 ± 8.6	HAMA SAS, CGI	Calligraphy
Zhao 2006 [18]	Patients with chronic schizophrenia	P: 48 C: 50	8 weeks	P: 25 ± 8.6 C: 24 ± 7.8	SCL-90	Calligraphy
Guo 2007 [19]	Schizophrenic inpatients	P: 30 C: 30	2 months	P: 31.3 ± 11.6 C: 32.3 ± 11.7	SAS, SDS	Music, calligraphy, painting, and dancing
Zeng 2007 [20]	Patients with generalized anxiety	P: 34 C: 34	8 weeks	P: 32.49 ± 8.2 C: 33.2 ± 8.3	HAMA SAS	Calligraphy
Zheng 2008 [21]	Patients with depression	P: 31 C: 30	6 weeks	P: 37.5 ± 12.5 C: 36.6 ± 12.8	HAMA HAMD	Calligraphy
Li 2010 [22]	Schizophrenic inpatients	P: 30 C: 30	8 weeks	P: 32.6 ± 11.3 C: 32.1 ± 11.2	BPRS SANS	Calligraphy and painting
Yang 2010 [12]	NPC patients	P: 24 C: 29	4 weeks	49.63 ± 10.81	SDS POMS SF	Three groups: relaxation, calligraphy, and control
Zhou 2010 [23]	Schizophrenic inpatients	P: 30 C: 30	3 months	40.38 ± 11.20	BPRS SANS	Calligraphy and painting
Kwok 2011 [13]	Older people with MCI	P: 14 C: 17	8 weeks	P: 85.79 ± 4.93 C: 85.76 ± 6.93	CMMSE	Calligraphy
Tian 2012 [24]	Patients with chronic schizophrenia	P: 60 C: 60	6 months	36.21 ± 2.54	BPRS	Occupational therapy, fine art, and calligraphy
Zhang 2012 [25]	Schizophrenic inpatients	P: 30 C: 30	12 months	–	PANSS	Calligraphy and painting
Dong 2013 [26]	Patients with chronic schizophrenia	P: 35 C: 34	12 weeks	P: 32 ± 7 C: 31 ± 9	SCL-90 PANSS	Calligraphy or drawing
Xu 2013 [27]	Undergraduate students	P: 8 C: 8	10 days	–	EEG-theta waves	Calligraphy
Zhou 2013 [28]	Grade 3 to 4 elementary school students	P: 101 C: 69	2 school years	P: 8.60 ± 0.55 C: 8.59 ± 0.41	PANAC-c CERO-k	Calligraphy
Zhu 2014 [29]	Children with hyperarousal symptoms	P: 64 C: 38	30 days	P: 10.52 ± 1.16 C: 10.54 ± 1.15	Salivary cortisol level	Calligraphy
		P: 129 C: 81		P: 10.51 ± 1.15 C: 10.52 ± 1.13	CRIES	
Tai 2016 [8]	Patients with AD	P: 14 C: 10	6 weeks	P: 70.21 ± 7.9 C: 76.3 ± 7.03	CDR-SB TC MMSE, GDS-S	Tai-chi, calligraphy, and drawing
Chan 2016 [30]	Individuals with MCI	P: 14 C: 16	8 weeks	P: 65.9 ± 5.0 C: 66.4 ± 3.65	Reaction time on the Chinese and digit 2-back and detection tasks	Calligraphy

*Abbreviations: SCL-90* Symptom Checklist 90, *CPQ* Children’s Personality Questionnaire, *PANSS* Positive and Negative Symptom Scale, *TESS* Treatment Emergent Symptom Scale, *HAMA* Hamilton Anxiety Scale, *SAS* Self-Rating Anxiety Scale, *CGI* Clinical Global Impression Scale, *SDS* Symptom Distress Scale, *HAMD* Hamilton Depression Rating Scale, *POMS SF* Profile of Mood State - Short Form, *BPRS* The Brief Psychiatric Rating Scale, *SANS* Scale for the Assessment of Negative Symptoms, *CMMSE* Chinese version of the Mini-Mental State Examination, *EEG* electroencephalogram, *PANAC-c* Positive and Negative Affect Scale for Children, *CERO-k* Child version of the Cognitive Emotion Regulation Questionnaire, *CRIES* Children’s Revised Impact of Event Scale, *CDR-SB* Clinical Dementia Rating Sum of Boxes, *TC MMSE* Traditional Chinese version of the Mini-Mental State Examination, *GDS-S* Geriatric Depression Scale, *SD* standard deviation, *NPC* nasopharyngeal carcinoma, *AD* Alzheimer’s disease, *MCI* mild cognitive impairment, *P* patient or experiment, *C* control, *p < 0.05, **p < 0.01

**Fig. 7:**
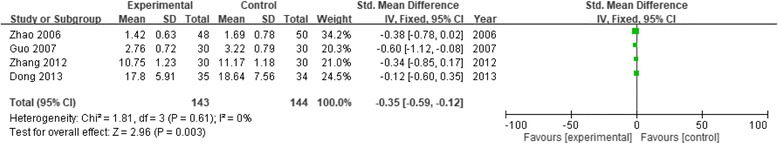
Forest plot of comparison: Experimental (Chinese calligraphy therapy, etc.) versus Control. Outcome: index of schizophrenia-psychopathy

**Fig. 8:**
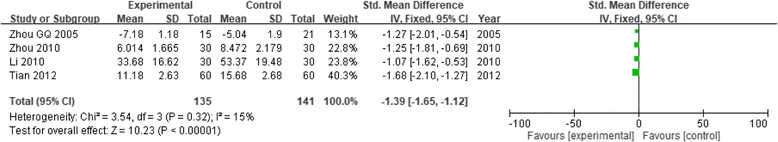
Forest plot of comparison: Experimental (Chinese calligraphy therapy, etc.) versus Control. Outcome: index of schizophrenia-negative syndrome

Eight studies evaluated patients with schizophrenia, of which four measured positive psychopathology of schizophrenia and four measured negative syndromes of schizophrenia (see Figs. 7 and 8 ).

Table 2 shows the methodological quality of the 21 included studies. Two studies clearly documented the use of random allocation [12, 13]. The remaining quality criteria were scored based on the narrative of the studies. The scores for risk of bias ranged from 6 to 9 points. Agreement between the two reviewers was assessed using Cohen’s kappa coefficient and was 0.891 (*p* < 0.001), indicating a high degree of consistency.

**Table 2:** Methodological quality assessment of the included studies (JBI)

Study	Study design	Score criteria										
		1	2	3	4	5	6	7	8	9	10	Total
Luo 2000	CCS	0	0	0	1	1	1	1	1	1	1	7
Cui 2003	CCS	0	0	0	1	1	1	1	1	1	1	7
Zhou B 2005	CS	0	0	0	1	0	1	1	1	1	1	6
Zhou GQ 2005	CCS	0	0	0	1	1	1	1	1	1	1	7
Dong 2006	RCT	1	1	1	0	1	1	1	1	1	1	9
Zhao 2006	RCT	1	1	0	1	1	1	1	1	1	1	9
Guo 2007	RCT	1	1	0	0	1	1	1	1	1	1	8
Zeng 2007	RCT	1	1	0	0	1	1	1	1	1	1	8
Zheng 2008	RCT	1	1	0	0	1	1	1	1	1	1	8
Li 2010	RCT	1	1	0	0	1	1	1	1	1	1	8
Yang 2010	RCT	1	1	0	1	1	1	1	1	1	1	9
Zhou 2010	RCT	1	1	0	0	1	1	1	1	1	1	8
Kwok 2011	RCT	1	0	0	0	1	1	1	1	1	1	6
Tian 2012	RCT	1	1	0	1	1	1	1	1	1	1	9
Zhang 2012	CCS	1	0	0	0	1	1	1	1	1	1	7
Dong 2013	RCT	1	1	1	0	1	1	1	1	1	1	9
Xu 2013	CCS	0	0	0	0	1	1	1	1	1	1	6
Zhou 2013	CS	0	0	0	1	0	1	1	1	1	1	6
Zhu 2014	RCT	1	1	0	0	1	1	1	1	1	1	8
Tai 2016	CCS	0	0	0	0	1	1	1	1	1	1	6
Chan 2016	RCT	1	1	0	0	1	1	1	1	1	1	8

*Abbreviations*: *JBI* Joanna Briggs Institute, *RCT* randomized controlled trial, *CCS* controlled clinical study, *CS* cohort study

Score criteria: 1. Was the assignment to treatment groups truly random? 2. Were participants blinded to treatment allocation? 3. Was allocation to treatment groups concealed from the allocator? 4. Were the outcomes of people who withdrew described and included in the analysis? 5. Were those assessing the outcomes blind to the treatment allocation? 6. Were control and treatment groups comparable at entry? 7. Were groups treated identically other than for the named interventions? 8. Were outcomes measured in the same way for all groups? 9. Were outcomes measured in a reliable way? 10. Was appropriate statistical analysis used?

Score descriptions: yes = 1, no = 0, unclear = 0, not applicable = 0

Figure 2 shows the meta-analysis results for group 1. Using a fixed-effects model, the heterogeneity test (I2) result for the seven studies was 0%, indicating homogeneity among the studies. The SMD was used to estimate the combined effect of measurements using different scales. The results showed that CCT can significantly reduce psychosis (10 studies, 965 subjects, SMD = − 0.17, 95% CI [− 0.30, − 0.04], Z = 2.60, *p* = 0.009).

Figure 3 shows the meta-analysis results for group 2. Using a fixed-effects model, the heterogeneity test (I2) was 0%, indicating that the nine studies were homogeneous. The SMD indicated that calligraphy treatment significantly reduced anxiety symptoms (9 studies, 579 subjects, SMD = − 0.78, 95% CI [− 0.95, − 0.61], Z = 8.98, *p* < 0.001).

Figure 4 shows the meta-analysis results for group 3. Using a fixed-effects model, the heterogeneity test (I2) was 0%, indicating that the seven studies were homogeneous. The SMD indicated that calligraphy treatment significantly reduced depressive symptoms (7 studies, 456 subjects, SMD = − 0.69, 95% CI [− 0.88, − 0.50], Z = 7.11, *p* < 0.001). Figure 5 shows the meta-analysis results for group 4. Using a fixed-effects model, the heterogeneity test (I2) was 0%, indicating that the two studies were homogeneous. The estimated combined effect showed that CCT significantly improved cognitive function (2 studies, 55 subjects, MD = 2.17, 95% CI [− 0.03, 4.38], Z = 1.93, *p* = 0.05). Figure 6 shows the meta-analysis results for group 5. Using a fixed-effects model, the heterogeneity test (I2) was 0%, indicating that the three studies were homogeneous. The estimated combined effect showed that CCT significantly improved neurofeedback (3 studies, 148 subjects, SMD = − 1.09, 95% CI [− 1.44, − 0.73], Z = 6.01, *p* < 0.001). Figure 7 shows the meta-analysis results for group 6–1 (schizophrenia-psychopathy). Using a fixed-effects model, the heterogeneity test (I2) was 0%, indicating that the four studies were homogeneous. The estimated combined effect showed that CCT significantly reduced positive psychopathological expression of schizophrenia symptoms (4 studies, 287 subjects, SMD = − 0.35, 95% CI [− 0.59, − 0.12], Z = 2.96, *p* = 0.003). Figure 8 shows the meta-analysis results for group 6–2 (schizophrenia-negative syndrome). Using a fixed-effects model, the heterogeneity test (I2) was 15%, indicating that the four studies were homogeneous. The estimated combined effect showed that CCT significantly reduced negative symptoms of schizophrenia (4 studies, 276 subjects, SMD = − 1.39, 95% CI [− 1.65, − 1.12], Z = 10.23, *p* < 0.001).

## Discussion

The purpose of this study was to explore the effectiveness of calligraphy therapy in improving symptoms of psychiatric disorders by reviewing and analyzing relevant literature. Twenty-one studies met the inclusion criteria and were reviewed. Most of the evidence suggests that CCT can change targeted behaviors in individuals with neuropsychiatric symptoms and that CCT is associated with improvements in objective measurements of psychiatric performance.

Evidence from these kinds of studies is needed before CCT can be considered effective for neuropsychiatric symptoms. The study findings reviewed here suggest that, used as psychiatric therapy, CCT can significantly improve selected neuropsychiatric symptoms. The use of CCT in compensation-focused interventions and selected psychotherapeutic interventions may lead to neuropsychiatric changes and thus improve daily life.

RCTs provide the best evidence of the efficacy of CCT. In RCTs with large samples, there is more balance between the characteristics of participants in the treatment and control groups. In small-sample RCTs, some characteristics may not be balanced between groups. Table 1 shows that most of the sample sizes in each group were between 15 and 30. Only about half of the included studies had quality scores greater than 8 (Table 2). Therefore, more high-quality RCTs are needed to strengthen the evidence for CCT’s effect in reducing neuropsychiatric symptoms. If more systematic reviews are produced to establish clinical guidelines, this could increase the clinical use of CCT.

A funnel plot of the CCT literature was symmetrical (i.e., showed no positive or negative relations between effect size and standard error). This indicates that there was no publication error. The best way to avoid publication bias is to begin with a rigorous examination of the literature.

As this is the first review of the efficacy of CCT for neuropsychiatric symptoms, we are unable to compare the findings with other similar studies. Compared with other non-pharmacological therapies like music therapy, painting therapy, or gardening therapy, CCT is perhaps a more culture-specific therapy, as it requires participants to learn Chinese writing and use a special soft calligraphy brush.

Our findings show that calligraphy therapy can significantly enhance cognitive function and relieve neuropsychiatric and depressive symptoms. However, there were some study limitations. First, studies varied markedly in their intervention approaches and selected outcome measures, and were frequently hampered by design limitations. Second, the pattern of effects on specific neuropsychiatric domains was inconsistent across studies. Additionally, some important outcomes, such as daily functioning, quality of life, and neuropsychiatric symptom severity, were assessed infrequently in the reviewed studies. Moreover, handwritten communication has largely been replaced by typed communications (and, in more recent years, by mobile phone calls and texts). This may have affected the results.

Overall, the results from trials are promising but inconclusive. Additional well-designed and adequately powered trials are warranted. However, this evidence must be treated with caution because of methodological limitations. To better assess the value of non-pharmacological interventions for this population, we recommend the following: (1) RCTs should have a large size of over 30 subjects; (2) standards for cognitive/neuropsychiatric rehabilitation must be established for treatment of diseases, such as stroke, Alzheimer’s disease and MCI; (3) general character templates for adaptation and modification in diverse fields of clinical trials must be standardized; (4) character form design must be set; (5) authority for standards of template designs, protocols for CCT and a large database for cases and trial follow ups must be established.

## Conclusion

This study shows that CCT exerts a curative effect on neuropsychiatric symptoms, but the evidence remains insufficient. A large number of RCTs are needed to facilitate additional systematic reviews of evidence for CCT. In general, we hope that this paper offers a method for a systematic CCT review and meta-analysis, which may provide a basis for establishing standards for CCT in clinical trials and applications in the future.

## Additional files

Additional file 1: AF1: Search terms (DOC 30 kb)

Additional file 2: List of studies excluded from the review by exclusion category (DOC 67 kb)
