# Supplementary material for: Does Chinese calligraphy therapy reduce neuropsychiatric symptoms: a systematic review and meta-analysis
Source: BMC Psychiatry. 2018 Mar 7;18:62. doi: 10.1186/s12888-018-1611-4 (PMC5842540; doi:10.1186/s12888-018-1611-4)
Supplement: Supplementary file 1 — AF1: Search terms (DOC 30 kb) [file 12888_2018_1611_MOESM1_ESM.doc]

**List of studies excluded from the review by exclusion category**

The following studies were excluded because they (1) were commentaries (n = 24) and (2) case reports (n = 4), (3) lacked a control group (n = 8), (4) had study populations that overlapped with other included studies (n = 6), and (5) were not intervention studies (n = 3).

The following list details the excluded studies based on the primary reasons for their exclusion.

**Criterion 1. Commentaries (n = 24)**

| 1 | Kao, HSR et al. Psychophysiological changes associated with Chinese calligraphy. Computer recognition and human production of handwriting. 1989; 349-81. |
| --- | --- |
| 2 | Gao SR; Miao MM. Hyperactivity-of-motion syndrome and behavioral correction and therapy. Calligraphy psychotherapy. 2000; 1:79. |
| 3 | Gao SR; Zheng Si-hong. Emotional Regulation of Calligraphy in Diabetic Patients. Calligraphy Psychotherapy. 2000; 1: 291. |
| 4 | Gao SR Liu I. Correction of Calligraphy Practice and NegativeBehavior. Calligraphy Psychotherapy. 2000; 1:145. |
| 5 | Ming, FEI. Traditional cultural instruments and artistic psychotherapy. Modern Rehabilitation. 2001; 21:008. |
| 6 | Gao, DG; Kao SR. Psycho-geometric analysis of commonly used Chinese characters. Cognitive neuroscience studies of the Chinese language. 2002; 195–206. |
| 7 | Kao, HSR; Goan, CH. Brush-writing instruments for health and therapy. U.S. Patent No. 6, 375, 622, 2002. |
| 8 | Kao, HSR, Shufa: Chinese calligraphic handwriting (CCH) for health and behavioural therapy. International Journal of Psychology. 2006; 41(4):282–86. |
| 9 | Zhang, J. et al. Chinese Calligraphy and Tourism: From Cultural Heritage to Landscape Symbol and Media of the Tourism Industry. Current Issues in Tourism. 2008; 11(6):529-48. |
| 10 | Hays, Pamela A. Integrating evidence-based practice, cognitive–behavior therapy, and multicultural therapy: Ten steps for culturally competent practice. Professional Psychology: Research and Practice, 2009, 40.4:354. |
| 11 | Kao, HSR. Calligraphy therapy: A complementary approach to psychotherapy. Asia Pacific Journal of Counselling and Psychotherapy. 2010. 1(1):55–66. |
| 12 | Kao HSR. Calligraphy health and calligraphy treatment. Applied Psychology. 2010, 46: 71–91. |
| 13 | Bao WY. The Effect of Calligraphy Practice Intervention on Promoting the Development of College Students' Positive Mood. Journal of Shangrao Normal College. 2010, 30.2: 117–120. |
| 14 | Wang ZR. The Role of Calligraphy in Psychotherapy. China Out-of-school Education: Middle. 2012, 1:16–16. |
| 15 | Ying YX. Theory and Practice of Painting and Calligraphy in Art Therapy. Journal of Huaihai Institute of Technology: Humanities and Social Sciences. 2012, 10.20:79–80. |
| 16 | Chan, CK. et al. Chinese talismans as a source of lead exposure. Hong Kong Med J. 2014, 20.4:347–9. |
| 17 | Yuan J; Dong J; Ding S. A Brief Analysis on the Promotion Effect of Calligraphy on Mental Health. Journal of Middle School. 2014, 10:213–213. |
| 18 | Cao JS. The Promotion of Calligraphy Practice on Mental Health of Senior High School Students. New Education (Hainan). 2015, 9:20–21. |
| 19 | Ming AH. Summarization of Calligraphy Psychotherapy, Science Bulletin. 2015; 17. |
| 20 | Jian D. Calligraphy can cure all diseases. Special health. 2015; 7:36. |
| 21 | Clark, Amanda CR. Contemporary Chinese Artists’ Books: New Artistic Voices in a Time of Transition. Art Documentation: Journal of the Art Libraries Society of North America. 2015; 34(1):15–28. |
| 22 | Shi Q. The Influence of Calligraphy Practice on College Students' Psychological Quality. Campus Psychology. 2016; 14(5):350-51. |
| 23 | Wang LY. The Relationship between Chinese Calligraphy and Physical and Mental Health. Master's Thesis. Ludong University. 2016 |
| 24 | Li J. Calligraphy art for children with ADHD treatment research. The new era of education (electronic magazine). 2016; 5:135–36. |

**Criterion 2. Case reports (n = 4)**

| 1 | Hu B. Analysis of Calligraphy Practice on Behavior Intervention in Mild Mentally Retarded Children. Chinese Special Education. 2005; 5:31–34. |
| --- | --- |
| 2 | Carneiro, Celeste. Art Therapy as a complementary therapy in the treatment of depression: a case study. Journal of Transpersonal Research. 2010; 2(1):62–72. |
| 3 | Liu BZ. Research on Adolescent Resilience and the Enlightenment to Post-disaster Psychological Rehabilitation. Journal of Xihua University (Philosophy & Social Sciences). 2011; 2:022. |
| 4 | Zhao GM; Wei LQ; Li K. Analysis of Short-term Psychological Counseling and Calligraphy Therapy for the Psychological Fatigue of the Coaches Intervening. Chinese Journal of Sports Medicine. 2013; 32(8):715–22. |

**Criterion 3. Lacked a control group (n = 8)**

| 1 | Liu Y. Calligraphy training on the mentally retarded children’s attention to the role of rehabilitation.1997. |
| --- | --- |
| 2 | Li ZQ, Yu Q, Zhang XQ Calligraphy behavior treatment of 63 patients with psychosis. China Rehabilitation. 2000; 15(3):3. |
| 3 | Wang YJ; Wang EY; Zou H. Effect of Calligraphy on Rehabilitation of Chronic Schizophrenia. 2001. PhD Thesis. |
| 4 | Liu JH; Liu WY. Analysis of effect of calligraphic drawing therapy on inpatients with mental illness. China Rehabilitation. 2003; 18(3):189–90. |
| 5 | Kao, HSR et al., Cognitive Facilitation Associated with Chinese Brush Handwriting: The Case of Symmetric and Asymmetric Chinese Characters. Perceptual and Motor Skills. 2004; 99 (3 suppl.):1269–73. |
| 6 | Hou DQ et al. The Adaptation Effect of Calligraphy and Music Therapy on Negative Emotions in Patients with Convalescent Schizophrenia. Chinese Journal of Rehabilitation Medicine. 2005; 10:766–67. |
| 7 | Wang CH. A study on application of multidimensional music and dance as artistic therapy. China's creative art “art and natural health.” 2009. |
| 8 | Zhou WX. Calligraphy as force of standing up. Geriatric Education (Senior University). 2015. |

**Criterion 4. Overlapped with other included studies (n = 6)**

| 1 | Zhou B; Liu JS; Zhou Y. Research on the Relationship between Calligraphy Practice and Children's Mental Health. Chinese Journal of Health Psychology. 2007; 15(5):434–36. |
| --- | --- |
| 2 | Zhou B; Liu JS; Liu BT. The Impact of Calligraphy Practice on Children's Multiple Intelligences Development. Psychological Science. 2010; 6:1509–11. |
| 3 | Zong Y. et al. Psychological intervention in post-traumatic stress response of 210 primary school students after calligraphy psychological therapy. Chinese Journal of Behavioral Medicine and Brain Science. 2010; 19(3): 267–68. |
| 4 | Zong Y. et al. Psychological intervention in calligraphic psychotherapy after traumatic stress response in children. Chinese Journal of Social Medicine. 2011; 28:31–33. |
| 5 | Yao N; Zhai AL; Li ZQ Study on the Effect of Calligraphy and Pain Rehabilitation Training on Schizophrenia Patients. International Journal of Nursing. 2011; 30.8:1260–62. |
| 6 | Zhou B. et al. The Promotion of Calligraphy Exercise on the Development of Children's Emotions and Their Regulatory Strategies. Psychological Science. 2013; 1:98–102. |

**Criterion 5. Not intervention studies (n = 3)**

| 1 | Lewinson TS. The use of handwriting analysis as a psychodiagnostic technique. Journal of Projective Techniques. 1961; 25:315–29. |
| --- | --- |
| 2 | Xin J. Study on the Influence of Calligraphy on Individual Mental Health and Quality of Life. PhD Thesis. Guangzhou University of Chinese Medicine. 2006. |
| 3 | Wang D; Zhang Y; Yao C. Stroke-based modeling and haptic skill display for Chinese calligraphy simulation system. Virtual Reality. 2006; 9(2)–3:118–32. |
