# Supplementary material for: Does Chinese calligraphy therapy reduce neuropsychiatric symptoms: a systematic review and meta-analysis
Source: BMC Psychiatry. 2018 Mar 7;18:62. doi: 10.1186/s12888-018-1611-4 (PMC5842540; doi:10.1186/s12888-018-1611-4)
Supplement: Supplementary file 2 — List of studies excluded from the review by exclusion category (DOC 67 kb) [file 12888_2018_1611_MOESM2_ESM.doc]

**AF1: Search terms**

***SEARCH 1: PubMed (conducted 21/12/16)***

1. Handwriting OR Chinese calligraphy OR Chinese calligraphy therapy OR Calligraphy exercise OR Calligraphy training [MeSH]

**AND**

2. Neuro- OR Neurology OR Neuropathy [MeSH]

**AND**

3. Psychiatric- OR Psychiatry [MeSH]

**Limits:** **Lack of Chinese literatures**

***SEARCH 2: PsycINFO (conducted 23/12/16)***

1. Handwriting OR Chinese calligraphy OR Chinese calligraphy therapy OR Calligraphy exercise OR Calligraphy training (Subject Heading)

**AND**

2. Neuro- OR Neurology OR Neuropathy (Subject Heading)

**AND**

3. Psychiatric- OR Psychiatry (Subject heading)

**Limits:** Lack of Chinese literatures

***SEARCH 3: Cochrane (conducted 27/12/16)***

1. Handwriting OR Chinese calligraphy OR Chinese calligraphy therapy OR Calligraphy exercise OR Calligraphy training [MeSH]

**AND**

2. Neuro- OR Neurology OR Neuropathy [MeSH]

**AND**

3. Psychiatric- OR Psychiatry [MeSH]

**Limits:** Lack of Chinese literatures

***SEARCH 4: Wanfang Data (conducted 29/12/16)***

1. Handwriting OR Chinese calligraphy OR Chinese calligraphy therapy OR Calligraphy exercise OR Calligraphy training (Subject Heading)

**AND**

2. Neuro- OR Neurology OR Neuropathy (Subject Heading)

**AND**

3. Psychiatric- OR Psychiatry (Subject Heading)

**Limits:** Less English literatures
